# Test–retest assessment of non-contrast MRI sequences to characterise and quantify the small bowel wall in healthy participants

**DOI:** 10.1007/s10334-021-00931-2

**Published:** 2021-06-05

**Authors:** Ali. S. Alyami, Hannah. G. Williams, Konstantinos Argyriou, David Gunn, Victoria Wilkinson-Smith, Jonathan. R. White, Jaber Alyami, Penny. A. Gowland, Gordon. W. Moran, Caroline. L. Hoad

**Affiliations:** 1 Faculty of Applied Medical Sciences, Diagnostic Radiology, Jazan University, Jazan, Saudi Arabia; 2 School of Medicine, University of Nottingham, Nottingham, UK; 3 Sir Peter Mansfield Imaging Centre, School of Physics and Astronomy, University of Nottingham, Nottingham, UK; 4 National Institute for Health Research (NIHR) Nottingham Biomedical Research Centre, Nottingham University Hospitals NHS Trust and the University of Nottingham, Nottingham, UK; 5 Diagnostic Radiology Department, Faculty of Applied Medical Sciences, King Abdulaziz University, Jeddah, Saudi Arabia

**Keywords:** Small intestine, Multiparametric magnetic resonance imaging, Validation study

## Abstract

**Objective:**

Quantitative Magnetic Resonance Imaging sequences have been investigated as objective imaging biomarkers of fibrosis and inflammation in Crohn’s disease.

**Aim:**

To determine the repeatability and inter- and intra-observer agreement of these measures in the prepared small bowel wall.

**Methods:**

Ten healthy participants were scanned at 3 T on 2 separate occasions using T1 and T2 relaxometry, IVIM-DWI and MT sequences. Test–retest repeatability was assessed using the coefficient of variation (CoV) and intra-class correlation coefficients (ICCs) were used to evaluate the intra- and inter-observer agreement

**Results:**

Test–retest repeatability in the bowel wall was excellent for apparent diffusion coefficient (ADC), magnetisation transfer ratio (MTR), T1, and diffusion coefficient D (CoV 5%, 7%, 8%, and 10%, respectively), good for perfusion fraction (PF) (CoV 20%) and acceptable for T2 (CoV 21%). Inter-observer agreement was good for the T2, D and ADC (ICC = 0.89, 0.86, 0.76, respectively) and moderate for T1 (ICC = 0.55). Intra-observer agreement was similar to inter-observer agreement.

**Discussion:**

This study showed variable results between the different parameters measured. Test–retest repeatability was at least acceptable for all parameters except pseudo-diffusion coefficient D*. Good inter- and intra-observer agreement was obtained for T2, ADC and D, with these parameters performing best in this technical validation study.

## Introduction

Magnetic Resonance Imaging (MRI) is now a gold-standard modality for non-invasively assessing disease activity and extent in small bowel Crohn’s disease (CD) [1, 2]. Multiple scoring systems have been set up to quantify the disease activity from T2-weighted and T1-weighted images [3–5]. These scoring systems rely on radiology observations which are subjective and time consuming to perform. Clinical T1-weighted and T2-weighted images cannot reliably differentiate fibrosis from inflammation in the bowel wall; an important determinant in decision-making for treatment options.

Recently, quantitative sequences such as diffusion-weighted imaging (DWI) [6, 7] and magnetisation transfer (MT) [8, 9] have been investigated as potential imaging biomarkers in CD. Intravoxel incoherent motion imaging (IVIM) [10] can provide contrast-free measures of tissue perfusion which is related to inflammation. MT reflects the exchange of magnetisation between protons in free water and protons bound to semisolid macromolecules and other moieties, and hence is a potential non-invasive measure of collagen deposition and intestinal fibrosis [8, 11–13]. Quantitative relaxometry T1 and T2 measures have shown potential as markers of fibrosis and inflammation in the liver [14, 15], pancreas [16, 17] and kidneys [18] and T2 of the small bowel wall has been shown to be related to intestinal permeability [19]. However, T1 and T2 measures have not been widely investigated in intestinal wall imaging.

These quantitative measures may provide an objective marker of disease activity which would be valuable in diagnosis, and monitoring progression and response to treatment [20]. As for most biomarkers, the validation of these imaging markers along the biomarker discovery roadmap is incomplete. In general, there are early and late phases to this roadmap [21], although there may be overlap between the two. The early phase is related to technical and biological validation which is rarely completed and which tend to be single site studies in small cohorts. Technical validation relates to repeatability, reproducibility and inter/intra-observer variability. Late phase downstream studies consider larger biological validation, clinical utility and clinical validation.

There is limited literature investigating the repeatability of these quantitative sequences in the small bowel. Moreover, oral preparation required prior to MR enterography (MRE) may have an effect on the signal intensity due to differential small bowel distension.

This study aimed to measure test–retest repeatability and inter/intra-observer agreement in T2, T1, IVIM-DWI and MT measurements of the small bowel wall following bowel preparation, and administration of an anti-spasmodic agent, in healthy participants on a single MRI platform.

## Materials and methods

### Study design

This was a single-centre prospective study recruiting healthy participants. The exclusion criteria included any history of gastrointestinal disease, pregnancy, contra-indications to anti-spasmodic agent usage, and any concomitant medication use that according to the investigators may affect gastrointestinal transit. The study was approved by the University of Nottingham Medical School Ethics Committee (J/3/2007/17) on 18/07/2017. Signed informed consent was obtained from all participants prior to recruitment. A subset of the T2 data has been reported previously [19].

**Fig. 1:**
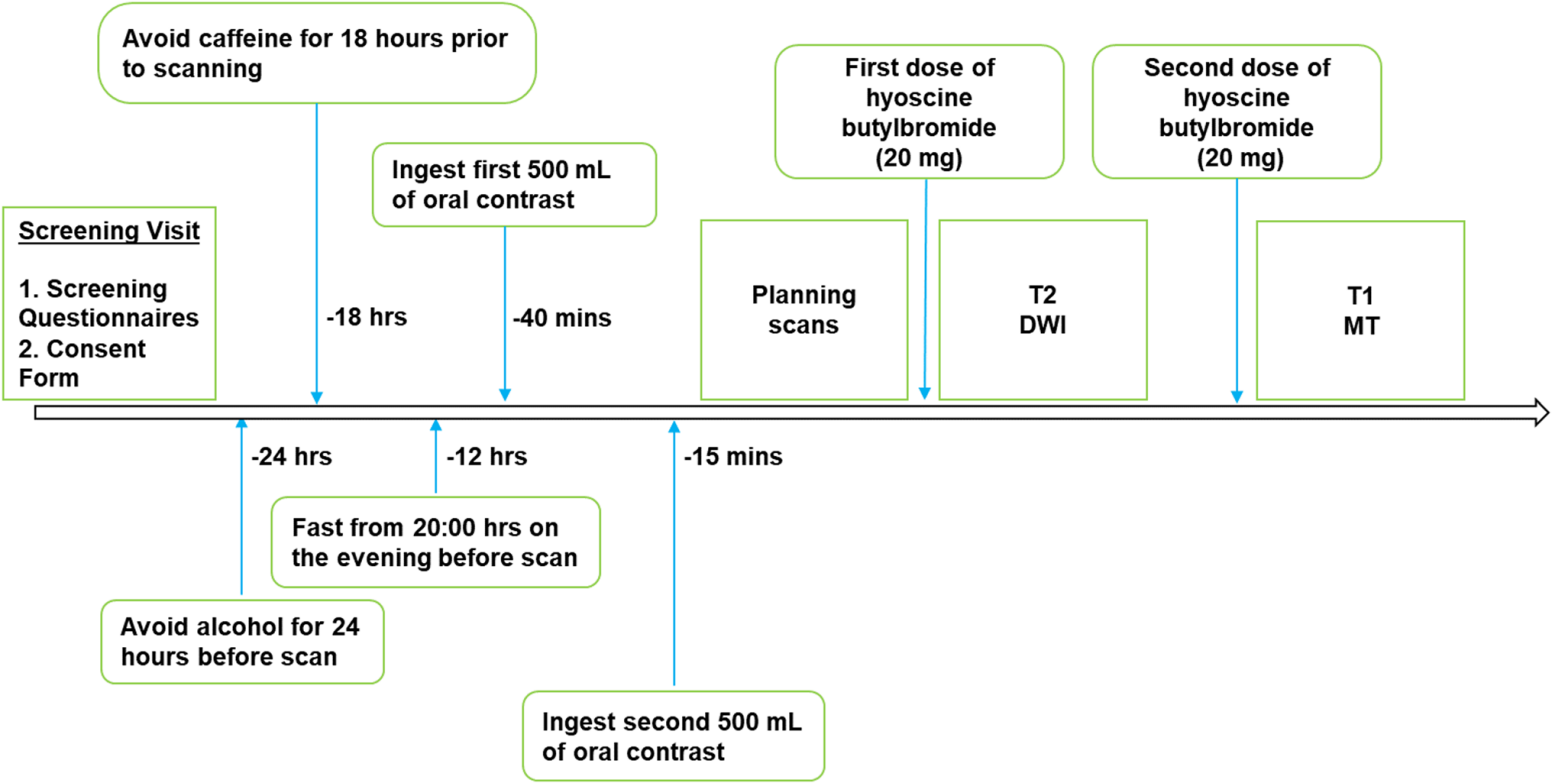
Protocol schematic for the repeatability study

Ten participants were scanned twice using an identical protocol, with a minimum of a two-week interval between visits. Participants were asked to fast from 22:00 h the previous evening, and to avoid ingesting caffeine and alcohol, and taking part in strenuous exercise the day prior to the study. Forty minutes before scanning, participants were given 1000 mL of bowel preparation (2.5% mannitol with 0.2% locust bean gum), to ingest slowly. After initial planning scans, and before the quantitative acquisitions, participants were given two separate doses of an anti-spasmodic agent (hyoscine butylbromide 20 mg) intravenously. One dose was administered before the T2 and DWI measurements, and the second dose before the T1 and MT sequence acquisitions (Fig. 1).

### MRI protocol

Imaging was performed using a 3 T Ingenia scanner (Philips, Best, The Netherlands) with participants positioned in a feet-first prone position. A respiratory belt was placed between the participant’s back and the MR coil using a velcro attachment to ensure a stable position, and a good respiratory trace for triggered scanning. Sagittal, axial, and coronal localisers were obtained to plan the position of the other sequences. The quantitative sequence information is given below with further details in Table 1.

- T2 data were acquired with a single slice coronal spin-echo prepared bTFE sequence [22], at six echo times (TE). The slice was positioned towards the anterior of the body to maximise coverage of small bowel loops.
- IVIM-DWI respiratory triggered data were acquired using a single-shot spin-echo echo-planar sequence (SE-EPI) at 9 b values, over 12 coronal slices, positioned to include as much of the small bowel as possible.
- T1 data were acquired from a single coronal slice using an inversion recovery prepared TFE sequence positioned in the same location as the T2 data. An additional image with no inversion was also acquired to provide a measure of the equilibirum magnetization.
- MT data were acquired using a saturation prepared (MT_on_) and standard (MT_off_) TFE scan, from a single coronal slice. The two scans were acquired during a single breath-hold of 11 s with a gap between the two dynamics of each scan. The off-resonance saturation was applied at 1000 Hz. Data from 2 different slices were acquired with one slice placed towards the anterior of the small bowel and the second towards the posterior

**Table 1:** Parameters of MRI scans

MRI parameters	T2 scan	T1 scan	IVIM-DWI scan	MT scan
Sequence	Single coronal slice spin-echo prepared bTFE	Single slice T1 weighted inversion recovery spoiled TFE	Coronal single-shot spin-echo-EPI	Single coronal slice TFE
Slice thickness (mm)	5	5	5	5
TR/TE (ms/ms)	3.4 / 1.68	10 / 2.3	1278 / 71	20 / 2.3
Field of view (mm^2^)	340 × 352	375 × 351	400 × 400	375 × 351
Number of slices	1	1	12	2
Breath-holding (BH)	1 BH per TE value with a 15 s gap between TE acquisitions	1 BH per TI value with a 15 s gap between TI acquisitions	Respiratory triggered	11 s BH per slice to cover both ‘on’ and ‘off’ resonance image acquisition
Parameter variation	TE-prep values: 20, 50, 80, 120, 180, 300 ms	TI values: 500, 650, 800, 950, 1000, 1150, 1300, 2500, 5000 ms	b-values: 0, 50, 100, 200, 300, 400, 600, 800, 1000 s/mm^2^	Presaturation MT on /off pulse
Approximate scan time including waiting between BH (mins)	2	3–4	4–6	1

### MRI Data analysis

All analysis was carried out using a different custom-written software for each sequence in MATLAB® (The MathWorks, Natick, MA, USA).

#### T2 Data

**Fig. 2:**
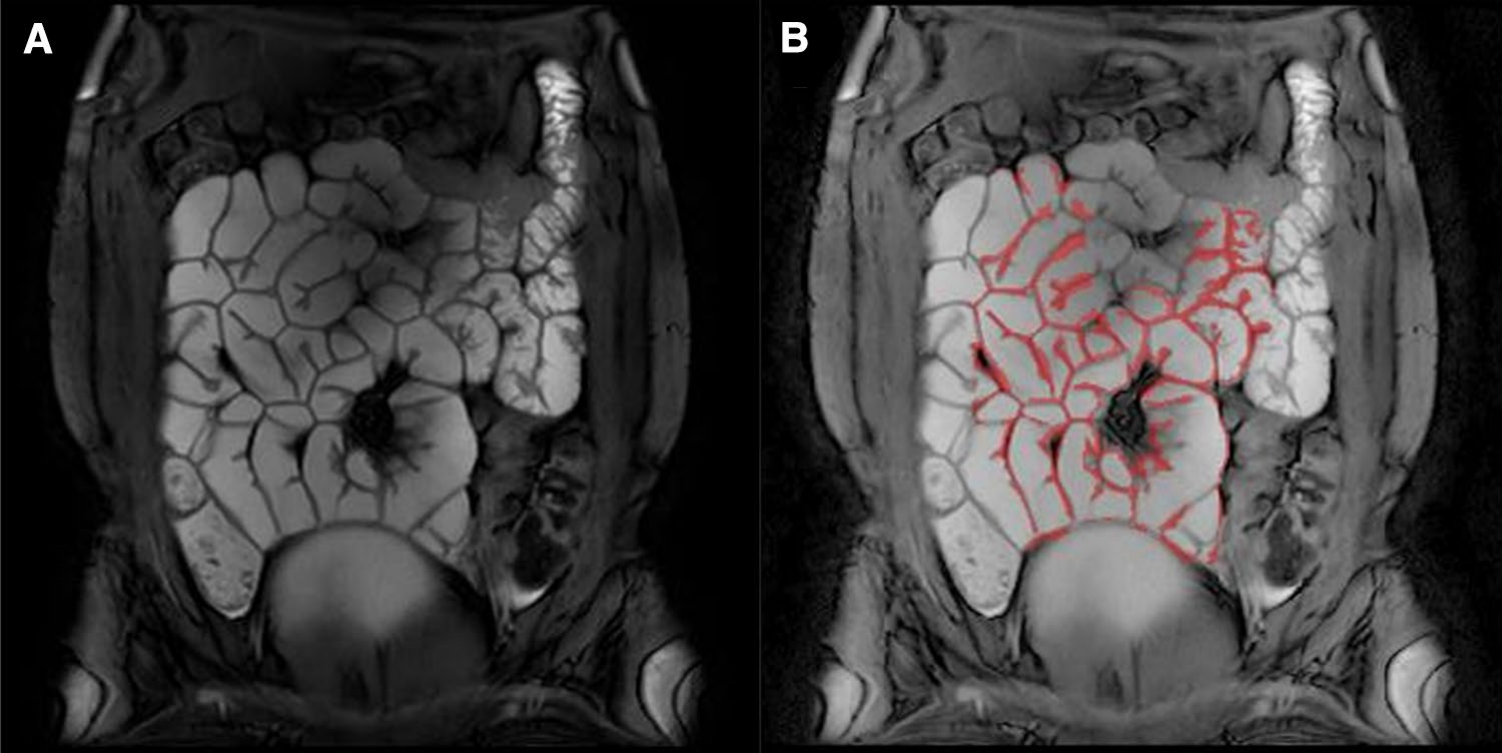
An example image highlighting the bowel wall that is automatically selected using a combination of thresholding and edge detection to define the walls. (A) T2 weighted bTFE image (TE = 20 ms). (B) Mask of the small bowel wall (red) overlaid on the TE = 20 ms image

The dataset was motion-corrected to remove distortions due to respiration and peristalsis [19] using a non-linear intensity-based motion correction algorithm in Matlab®. The analysis was performed using a semi-automated program which used edge detection and thresholding to isolate the bowel wall [19]. The wall was then split into multiple ROIs and the signal from each ROI fitted for T2 taking account of the effect of the full bTFE readout [22]. A two-compartment model was used to overcome partial volume effects (small bowel wall and content). Figure 2 shows an example of the small bowel mask created using the software. The observer interacted with the software for approximately 2 min per subject study visit to generate the ROIs signal for fitting. The automatic motion correction section of the algorithm took around 5 min to run per subject study visit.

#### T1 Data

ROIs were drawn along the bowel on the shortest inversion recovery image. These ROIs were then applied to the remaining images and moved rigidly to account for any motion that occurred throught the aquistion. ROIs were also drawn in the contents of the bowel. First, the mean signal from the content ROIs was fitted to an inversion recovery model including a parameter to take account of the degree of inversion (α), using the least-squares non-linear curve-fitting algorithm in Matlab®. Next, the wall data were fitted to a similar two compartment inversion recovery model, but this time including the T1 and α for the contents from the previous fit, to allow for partial volume effects in the ROI to be taken into account. The process was repeated for several bowel wall ROIs, with the aim to define a minimum of 10 different ROIs per image. The time taken to complete each ROI (including fitting the data) was between 2 and 5 min depending on how much motion correction to the ROI was needed.

#### MT Data

**Fig. 3:**
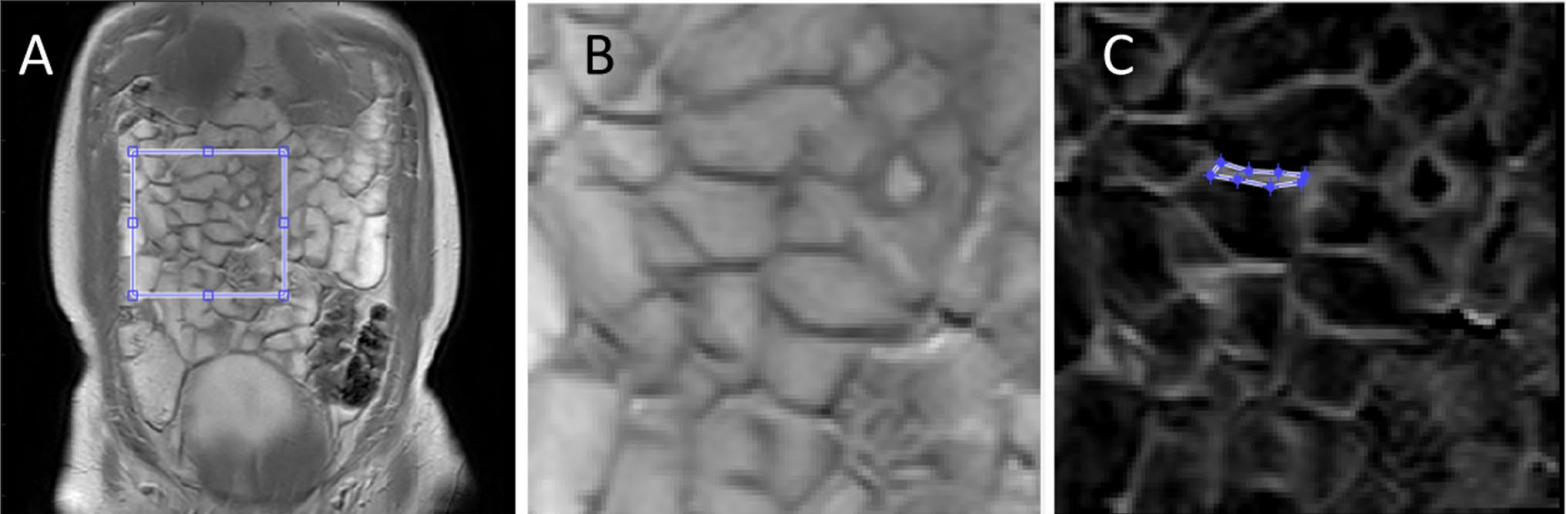
An exmaple of ROI definition on MT images. The ROI was drawn on the small bowel wall after motion correction of the images. **a** shows the raw MT-on image with cropped area highlighted which was the target for motion correction.** b** Cropped area after motion correction. **c** MTR image with the bowel wall ROI highlighted

MTR values were determined from ROIs of the mean signal intensity in the MT data. ROIs were defined using custom-written software in Matlab® following several steps. First, a region of the image containing small bowel was selected (Fig. 3a) and rigid-body motion correction was applied between the MT_on_ and MT_off_ data for that region using an intensity-based algorithm. An MTR map (MT_on_-MT_off_)/MT_off_ was calcuated across the region. The MTR map was used to determine whether the small bowel wall had visually improved after the motion correction applied. Improvement was defined as the wall edges being sharper and the walls thinner visually on the MTR image. Obvious wall movement between the on and off data was also checked visually. If large scale motion, seen between the on and off original data, was not corrected using motion correction, a new region was selected. Motion correction was not always optimal due to the change in contrast between the bowel wall and contents across the two different MT images. If the images (either original data or motion corrected) were deemed acceptable for analysis, the ROI was then drawn on the selected MTR image. The MTR value calculated for the ROI was the median of the data. This process was repeated multiple times to acquire multiple regions. An example showing the MT ROI definition is shown in Fig. 3. The time taken to complete each ROI (after the motion correction which took approximately 1 min) was between 1 and 2 min.

#### IVIM-DWI Data

Initially, an intensity-based rigid-body motion correction was run on a small section of the image containing small bowel; however, if this did not visually improve the alignment of the data and no obvious wall motion was present, then the raw data images were used to define the ROIs. An ROI was drawn on the bowel wall, and was moved rigidly between different b-value images if necessary. The mean signal intensity values from all b-value data were then calculated from the ROI, and this process was repeated multiple times for different bowel wall ROIs. A two-step process was used to fit the data [23] to the IVIM [24] equation given below

1 $${\text{S}}_{{\text{i}}} /{\text{S}}_{0} \, = \,{\text{PF}}*{\text{exp}}\left( { - {\text{b}}_{{\text{i}}} \left( {{\text{D}}*\, + \,{\text{D}}} \right)} \right)\, + \,\left( {{\text{1}} - {\text{PF}}} \right){\text{ exp}}\left( { - \,{\text{b}}_{{\text{i}}} {\text{D}}} \right)$$

where S_i_ is the signal intensity for b-value b_i_, S_0_ the signal intensity for b = 0 s/mm^2^, PF is the perfusion fraction, D the tissue diffusion coefficient and D* the pseudo-diffusion coefficient (related to capillary perfusion). First, the diffusion coefficient (D) and and perfusion fraction (PF) were estimated using data with b-values larger than 200 s/mm^2^, from the mono-exponential fit of

2 $${\text{S}}_{{\text{i}}} /{\text{S}}_{0} \, = \,\left( {{\text{1}} - {\text{PF}}} \right){\text{ exp}}\left( { - \,{\text{b}}_{{\text{i}}} {\text{D}}} \right)$$

as at high b-values the first term in Eq. (1) approximates to zero. Second, these results for D and PF were used as the initial estimate of those parameters in a non-linear fit to the full equation, which also evaluates D* using data from all b-values [10]. The time taken to complete each ROI (including fitting the data) was between 2 and 5 min depending on how much motion correction to the ROI was needed. The rigid body motion correction also took around 3–5 min to run for each small section.

Test–Retest repeatability and inter-observer and intra-observer agreement analysis.

The participants were scanned twice in two weeks to assess repeatability. Two observers independently anaylsed the MT, T2 and T1 (AA—2 years SB MRI experience, HW—3 years SB MRI experience) and IVIM-DWI (AA, CH—10 + years SB MRI experience) data. Results from observer AA were used to evaluate test–retest repeatability. The results from both observers were used to evaluate inter-observer agreement. Both observers then repeated the measurements for intra-observer agreement, a minimum of three months seperated the repeated analysis.

### Statistical analysis

For all the parameters measured, the median value across all ROIs drawn from each visit or analysis were used for the subsequent statistical analysis. For descriptive statistics, the data were assumed to be non-parametric due to the small number of participants in the study and data were expressed as median, and interquartile range (IQR).

The coefficient of variation (CoV%) was used as a measure of test–retest repeatability. The CoV % was computed as the percentage of the standard deviation (SD) of the mean (SD*100%/mean) calculated from Visit 1 and Visit 2 data for each participant individually, and then averaged across all participants. The repeatability of each parameter was defined as poor when CoV was > 30%, acceptable when CoV was between 20 and 30%, good when CoV was between 10 and 20%, and excellent when CoV ≤ 10% [25]. To assess the spread in the data across all the participants (biological variation), a CoV was measured across Visit 1 (observer 1) data from all participants (sd across all Visit 1 participants’ data*100%/mean across all Visit 1 participant data). This across-participant data were compared with the within-participant data to determine whether the test–retest variability was lower than the variation seen across participants.

The intra and inter-observer agreement was assessed by calculating the intra-class correlation (ICC) with a two-way mixed model of absolute agreement and was interpreted as follows: values less than 0.5 were indicators of poor agreement, values between 0.5 and 0.75 were indicators of moderate agreement, values between 0.75 and 0.9 were indicators of good agreement, and values greater than 0.90 indicated excellent agreement [26]. In addition to the CoV and ICC calculations, Bland–Altman plots were used to assess agreement for the test–retest data and observer measurements[27, 28].

Statistical analyses were performed using SPSS version 25 (IBM Armonk, NY) or GraphPad Prism version 8.0 for Windows (GraphPad Software, La Jolla California USA).

## Results

### Participants’ characteristics

**Fig. 4:**
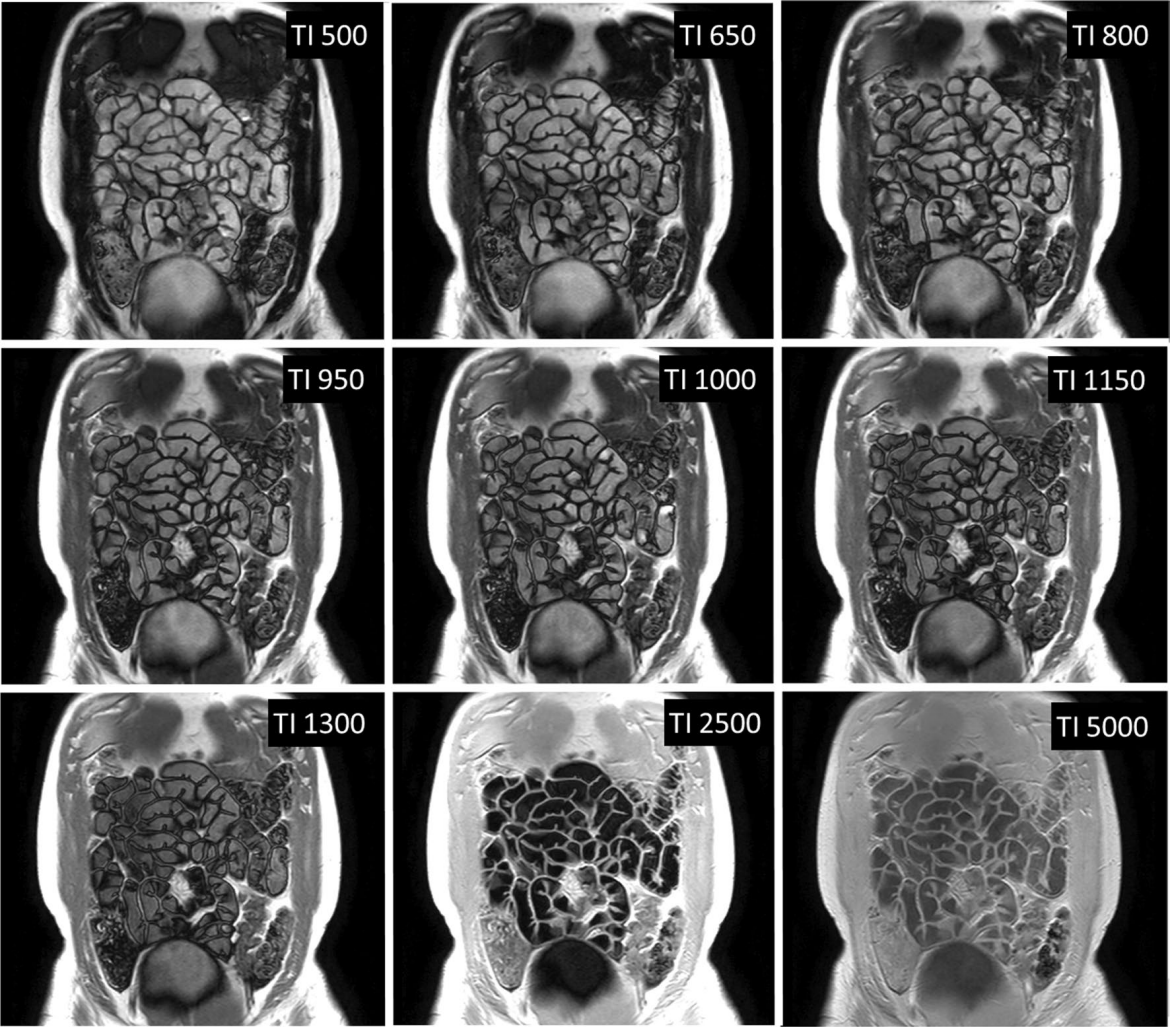
Single slice coronal images of the abdomen showing the T1 recovery of the bowel wall with different TIs in ms

**Fig. 5:**
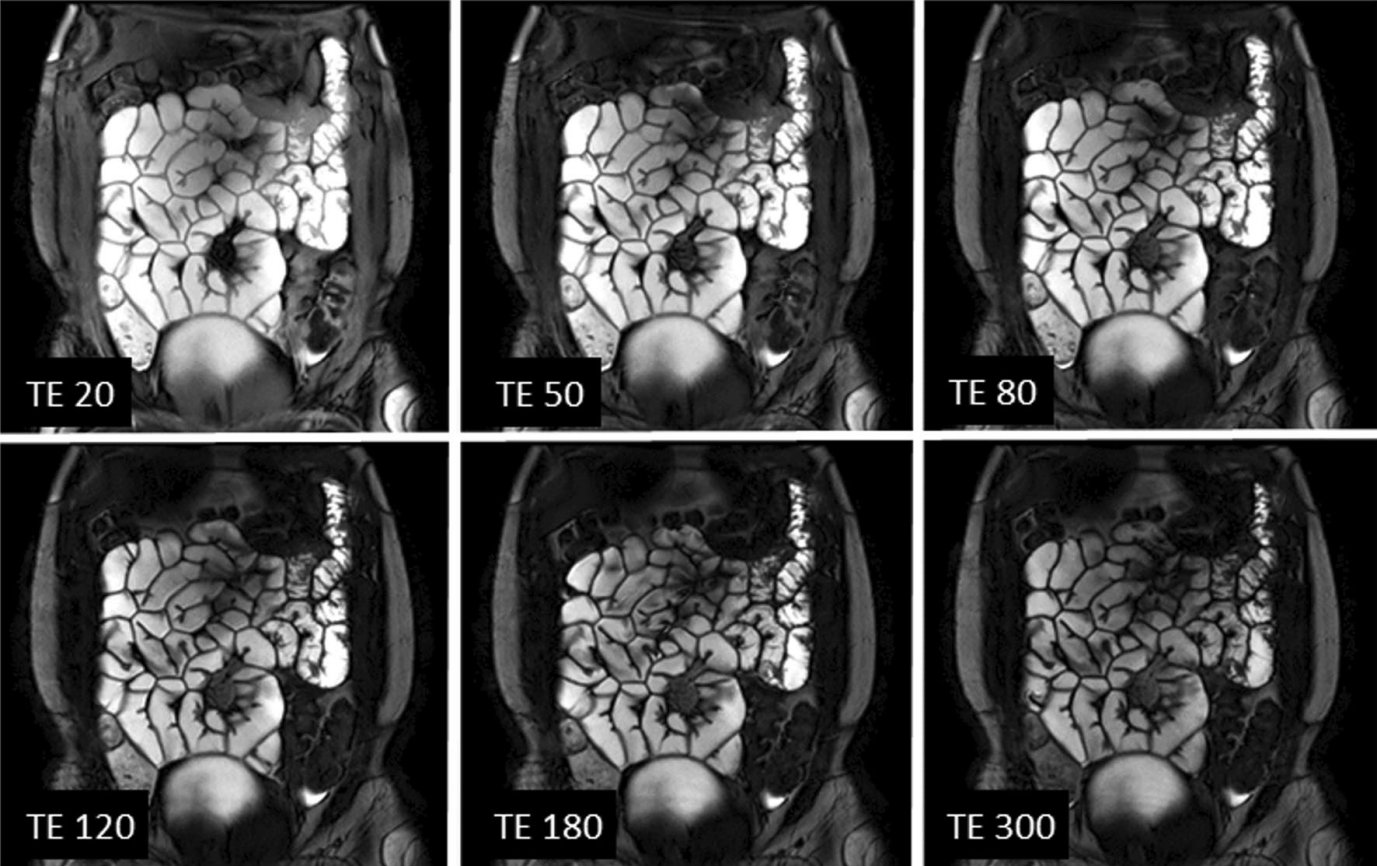
Single slice coronal image of the abdomen showing T2 decay of the bowel wall with different TEs in ms

Ten participants were screened and recruited. All participants (9 female, 1 male: mean age 30 ± 8 yrs) completed both scans. Two participants were excluded from T2 data analysis by both observers due to observed through plane motion which caused the bowel to move out of the imaging slice during the acquistion. One participant was removed from the T1 analysis due to the same reason from one observer, and four from the second observer. Figures 4 and 5 show example images for T1 and T2 data, respectively.

### Test–retest repeatability

**Fig. 6:**
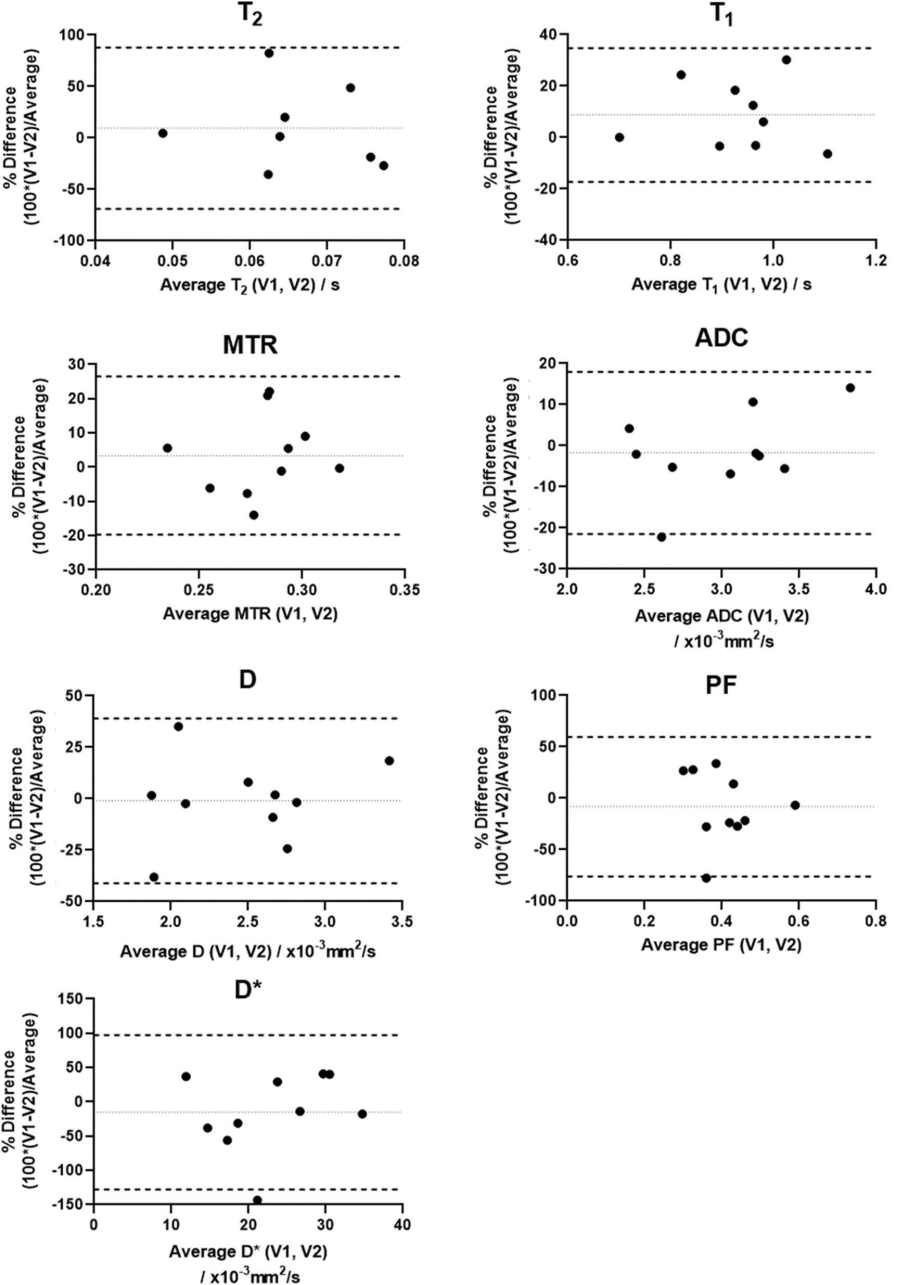
Bland–Altman plots showing test–retest repeatability (% Difference vs average) for all measured quantitative parameters calculated from the small bowel MRI scans. Dotted lines indicate the bias (central line) and Dashed lines the corresponding 95% limits of agreement

Table 2 shows that the test–retest repeatability from the CoV analysis was excellent for ADC, MTR, T1 and D, good for PF, acceptable for T2, and poor for D*. Graphic illustration of the test–retest repeatability using Bland–Altman plots are shown in Fig. 6, with limits of agreement less than 30% of average for MTR and ADC, between 30 and 50% of average for T1 and D and greater than 50% for T2, PF and D*.

**Table 2:** Test–retest repeatability, mean coefficients of variation (CoVs), Bland–Altman limits of agreements (BA-LA) and Intra-class correlation coefficients (ICC) of T2, T1, MTR, ADC, D, PF and D* parameters. CoVs across all participants calculated from Observer 1 Visit 1 data is also presented to indicate the variations in the parameters across the different participants

Test–retest (within-participant)							Across participants Variation
Parameter	N	Visit 1 Median (IQR)	Visit 2 Median (IQR)	CoV %	BA bias [LA]	ICC, p value (95% CI)	CoV %
T2 (s)	8	0.067 (0.054–0.083)	0.060 (0.049–0.080)	21	0.006 [−0.046–0.057]	− 0.385, p = 0.818 (−0.978 – 0.445)	21
T1 (s)	9	1.0 (0.90–1.0)	0.90 (0.77–0.96)	8	0.08 [−0.16–0.32]	0.485, p = 0.05 (−0.095 – 0.846)	13
MTR	10	0.29 (0.25–0.31)	0.28 (0.25–0.29)	7	0.01 [−0.05–0.07]	0.337, p = 0.157 (−0.370 – 0.779)	11
ADC (10^–3^ mm^2^/s)	10	3.06 (2.44–3.32)	3.06 (2.67–3.33)	5	-0.03 [−0.63–0.57]	0.814, p = 0.002 (0.409 – 0.901)	18
D (10^–3^ mm^2^/s)	10	2.4 (2.02–2.72)	2.5 (2.05–2.89)	10	0.01 [−0.93–0.91]	0.650, p = 0.020 (0.042 – 0.901)	24
PF	10	0.37 (0.32–0.45)	0.43 (0.30–0.50)	20	-0.03 [−0.28–0.20]	0.273, p = 0.211 (−0.385 – 0.750)	24
D* (10^–3^ mm^2^/s)	10	20 (12–32)	22 (19–30)	31	−2.5 [−27–22]	0.178, p = 0.310 (-0.522 – 0.712)	50

The within participant test–retest CoV data showed lower variability when compared to the CoV measured across all participants at visit 1 for all parameters except T2.

### Inter-observer agreement

**Fig. 7:**
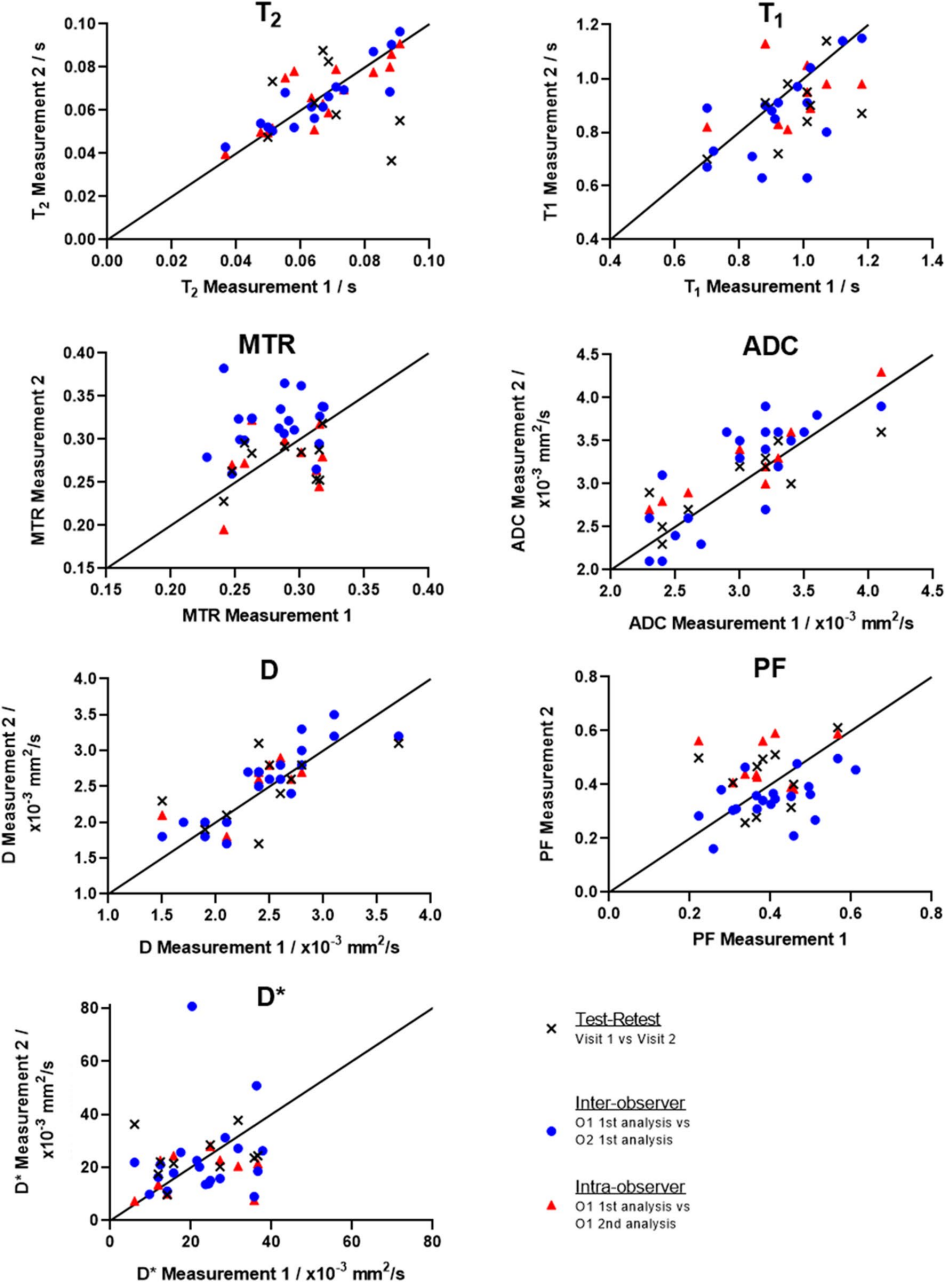
Correlation graphs for all the measured MRI parameters showing the Test–Retest, Inter- and intra-observer data

Table 3 shows the ICC for the inter-observer variability, along with the Bland–Altman limits of agreement. The median number of ROIs used to generate each measurement are also presented. Inter-observer variability was good for T2, D and ADC, moderate for T1 and poor for MTR, PF and D*. The correlation graphs for this data are shown in Fig. 7.

**Table 3:** Inter- and Intra-observer agreement intra-class correlation coefficients (ICCs) and Bland–Altman (BA) Bias and limits of agreements (LA) of the measured parameters. The number of measurements for each set of data is given by N. The median (IQR) number of ROIs used for each measurement are also given

Inter-observer Data: Observer 1 and Observer 2					
Parameter	*N*	Median (IQR) Observer 1	Median (IQR) Observer 2	ICC [95% CI]	BA Bias [LA]
T2 (s)	16	0.065 (0.052–0.080)	0.063 (0.052–0.070)	0.89 [0.71–0.96]	0.004 [−14–15]
Number of ROIs for T2	16	86 (51–165)	105 (68–177)		
T1 (s)	16	0.91 (0.84–1.01)	0.88 (0.71–0.95)	0.55 [0.13–0.81]	0.06 [−0.20–0.33]
Number of ROIs for T1	16	12 (10–12)	10 (10–10)		
MTR	20	0.29 (0.25–0.31)	0.32 (0.30–0.34)	0.08 [−0.15–0.39]	−0.04 [-0.11–0.04]
Number of ROIs for MT	20	18 (13–26)	29 (25–32)		
ADC (10^–3^ mm^**2**^/s)	20	3.1 (2.52–3.30)	3.35 (2.6–3.60)	0.76 [0.49–0.9]	−0.13 [−0.84–0.57]
D (10^–3^ mm^2^/s)	20	2.45 (2.10–2.80)	2.65 (2.0–2.95)	0.86 [0.68–0.94]	−0.09 [− 0.62–0.43]
PF	20	0.40 (0.32–0.48)	0.35 (0.30–0.38)	0.41 [0.01–0.71]	0.05 [− 0.13–0.24]
D* (10^–3^ mm^**2**^/s)	20	22 (14–30)	19 (14–26)	0.14 [−0.33–0.54]	−0.6 [− 35–34]
Number of ROIs for DWI	20	5 (4–7)	13 (11–15)		
Intra-Observer Data: Observer 1 Only					
Parameter	*N*	Median (IQR) first measurement	Median (IQR) second measurement	ICC [95% CI]	Bias BA-LA
T2 (s)	8	0.067 (0.054–0.083)	0.060 (0.051–0.084)	0.91 [0.65–0.98]	0.002 [− 0.010–0.015]
T1 (s)	9	1.0 (0.90–1.0)	0.95 (0.8–2-1)	0.33 [− 0.40–0.80]	0.033 [− 0.24–0.31]
MTR	10	0.29 (0.25–0.32)	0.28 (0.26–0.30)	0.32 [− 0.36–0.77]	0.01 [− 0.07–0.9]
ADC (10^–3^ mm^2^/s)	10	3.06 (2.44–3.32)	3.10 (2.8–3.45)	0.85 [0.18–0.96]	− 0.21 [− 0.62–0.20]
D (10^–3^ mm^2^/s)	10	2.4 (2.02–2.72)	2.6 (2.07–2.82)	0.83 [0.47–0.95]	− 0.08 [− 0.72–0.56]
PF	10	0.37 (0.32–0.45)	0.43 (0.40–0.56)	0.05 [− 0.31–0.55]	− 0.09 [− 0.33–0.14]
D* (10^–3^ mm^**2**^/s)	10	20 (12–32)	21 (12–32)	0.22 [−0.42–0.72]	3.8 [− 19–26]

### Intra-observer agreement

Table 3 also shows the ICC for the intra-observer agreement from 2 separate measurements of the data. Intra-observer ICC agreement for observer 1 was excellent in the bowel wall for T2, good for D and ADC and poor for T1, MTR, PF and D*. Observer 2’s data produced similar results (data not presented). Correlation data are shown graphically in Fig. 7.

## Discussion

The main goal of this study was a technical validation of MRI-based quantitative measures of T1 and T2, MTR and IVIM-DWI in the small bowel wall on a 3 T MRI platform. Test–retest data provide a measure of how repeatable the test situation and subsequent analyses are to generate a stable measurement of interest, whereas inter- and intra-observer agreement provide data on the variability in a single observation for one or multiple observers. For this study, all healthy participants were studied on the same MRI scanner by the same experimental team following the same protocol. We have shown that the test–retest repeatability CoV was excellent for ADC, MTR, T1 and D, good for D, and acceptable for T2, but poor for D*. In addition the Bland–Altman limits of agreement were less than 30% for MTR and ADC, but were more than 50% of the average for T2, PF and D*. Regarding the inter-observer agreement, ICC was good for T2, D and ADC, moderate for T1, while poor for MTR, PF and D*. Similar intra-observer variability for T2, D and ADC was good to excellent, while poor for MTR, PF and D*. However, ICC is partly dependent on the dynamic range of the data measured; with small dynamic ranges potentially showing less correlation;T1 and MTR had variations of 11 and 13% across participants, the lowest of all parameters measured.)

Test–retest relaxometry data in other organs have shown smaller CoV data, with T1 in the liver at 1.8% [15] and kidney 2% [29]. T2 in the extraocular muscles was found to be of the order of 2–5% [30]. These are all considerably lower than our data. However, whole organ measurements (liver and kidney) are expected to be much more repeatable data since it is easier to define ROIs in larger organs and these are all less sensitive to small movements. Furthermore, it will be easier to ensure the same region is sampled between scans in the larger organs, which was difficult here, where a single slice was acquired across the bowel wall so that different parts of bowel may have been sampled for the test–retest study. In the cervical spinal cord, Levy et al. [31] showed poor ICCs for MTR ranging from -0.3 to 0.4 for different vertebral levels for their test–retest data from 16 subjects, whereas the data from our study were at the upper end of this range at 0.3. The IVIM-DWI measures showed similar repeatability when compared to other organs, with ADC and D having lower CoV compared to PF and D*. Liver hepatocellular carcinomas produced test–retest CoVs of 15.6% and 19.7% for ADC and D, respectively [25], higher than those measured in this study. However, similar values to this study were found in the kidney [29], with 2.9%, 9.5% and 39% for ADC, D and D* respectively, compared to 5, 10 and 31% for the same paramaters in the bowel wall. These results may reflect a better multi-slice sampling across the whole bowel used for DWI compared to the other quantitative measures.

In terms of inter and intra-observer agreement, T2, which used the most automated procedure for ROI definition, produced the highest agreement between measurements. MTR required manual definition of the ROIs which increased variability and sensitivity to partial volume effects that could not be overcome in the same way as for T1 and T2 meaurments (which used two compartment fits). In addition, poor breath-holding and bowel motion would introduce more noise into the 2-point measurement, which may have been smoothed out if using a curve-fitting approach. Variation in B1 and B0 field effects could also influence the measured MTR; these problem could be addressed somewhat in future by acquiring data at a variety of saturation powers and frequency offsets (z-spectra). ADC and D showed good observer agreement, but PF and D* showed poor agreement, which is similar to previous studies in the liver [25] and may reflect the limited number of low b-values used [32].

Other studies have measured some of these quantitative measurements in the small bowel wall and the data from this study agree well with the previously published literature for T2 and MTR. The placebo arm of a healthy volunteer provocation study [19] measured the T2 of the bowel wall in 16 subjects with a mean (sd) of 0.070 (0.036) s compared to this study which measured a median 0.067 s from the 8 volunteers’ visit 1 data. MTR was measured in both fibrotic and healthy appearing tissue from Crohn’s disease patients by Pazahr et al. [11]. They measured a mean (sd) MTR of 25.4 (3.4)% in the healthy tissue compared to 35.3 (4.0) % in the fibrotic tissue. The range of the MTR measured in healthy appearing tissue was large (17–32%) [11], with the data from this study having a median of 29% and smaller total range of values across both visits (23–32%), which may be due to the multiple ROI measurements carried out for this study reducing the overall variability. Several studies have investigated IVIM parameters in normal or non-fibrotic appearing bowel wall in Crohn’s disease patients [6, 33, 34]. The median ADC, D and D* measured in this study were slightly higher than those measured in Crohn’s studies (ADC 2.7 (0.5) and D 1.7 (0.7) × 10^−3^mm^2^/s of Freimann et al. [33], with slightly lower values recorded by Hectors et al. [6]). This may reflect some partial volume effects of the luminal content from the thin bowel wall in healhty volunteers, along with differences in b-values used to calculate the parameters. The range and median PF values measured in the different studies [6, 34] were similar when compared to this study (median/mean values around 0.4). To date, there have been no previous publications of measuring T1 in the bowel wall.

All quantitative measurements in the bowel present technical challenges. The bowel must be distended using oral contrast, which can result in uneven distension of the wall influencing the MRI parameters measured. Bowel motion must be eliminated for accurate quantification, which limits the maximum experimental length due to the short biological half-life of anti-spasmodics available [35]. This limits the amount of data that can be acquired for quantification (number of TI/TE/b-values used, coverage across the bowel), which will influence the overall repeatability of the measurement. Thin slices, to reduce partial volume effects across the walls also increase the likelihood of through plane motion further degrading the data.

The technique of magnetic resonance fingerprinting allows for rapid simultaneous measurements of T1 and T2 [36]. This technique was developed in the brain, and due to additional challenges of large scale B1 and B0 inhomogeneties and respiratory motion, has only recently been applied in the abdomen and pelvis [37–40]. Promising results for solid organs (e.g. liver, kidney [37], pancreas [39]) have been obtained; however, it has yet to be applied in the bowel. This may be because in-plane resolution for adequate bowel wall delineation may be difficult to achieve during the breath-hold scans currently required. Higher resolution and combined diffusion scans have been applied in the pelvis where breathholding is not an issue [40].

Our study had some limitations. We included a relatively small number of participants to this study due to the need for oral bowel preparation and administration of anti-spasmodics. Some data were also lost due to through-plane motion of the bowel between the different acquisitions of the T1 and T2 data and as the decision to reject data for motion was an observer task for T1 there were discrepancies between the observers as to which data to exclude. A 3D approach to data acquisition and a more automated approach to identify large scale motion would reduce the amount of data with these errors and discrepancies between observers. Most of the data analyses involved manual drawing of the ROIs, which added noise to the measurements from both the individual ROI defined and the placement of the ROIs along the bowel wall. These two factors probably provided most of the differences between the 2 observers. Only the IVIM data were derived from a large number of slices covering the majority of the small bowel. This was not the case in T2, T1 and MTR, and so the results are not directly comparable between measures. Expanding the measurements to multiple slices or 3D acquisition would be one way to overcome this particular limitation. The performance of some of these measures may be improved in diseased tissue where wall thickness is increased and motility reduced.

In conclusion, our study assessed the repeatability of 7 quantitative parameters in the assessment of small bowel walls and measured the observer agreement. In the bowel wall, test–retest repeatability was excellent for ADC, D, MTR, T1, good for PF, and acceptable for T2, but was poor for D*, whereas the inter- and intra-observer agreement was good for T2, ADC and D. Overall, T2, ADC and D performed best for the methods and scan times used in this technical validation study. Further studies are needed to investigate the aetiology of the changes observed in these parameters to fully understand their role and potential use in the small bowel wall. Moroever, 3D or multiple slice imaging and more automated analyses will invariably decrease bias and variability within these readouts.

## Data Availability

Data available on request due to privacy/ethical restrictions.

## Abbreviations

ADC: Apparent diffusion coefficient
CD: Crohn’s Disease
CV: Coefficient of variation
D*: Pseudodiffusion coefficient
D: ‘True’ diffusion coefficient
DWI: Diffusion-weighted imaging
ICC: Intra-class correlation coefficient
IVIM: Intravoxel incoherent motion
MRI: Magnetic Resonance Imaging
MTR: Magnetization transfer ratio
PF: Perfusion fraction
ROI: Region of interest
